# Sex-specific impact of diabetes mellitus on left ventricular systolic function and prognosis in heart failure

**DOI:** 10.1038/s41598-021-91170-x

**Published:** 2021-06-03

**Authors:** Soongu Kwak, In-Chang Hwang, Jin Joo Park, Jae-Hyeong Park, Jun-Bean Park, Goo-Yeong Cho

**Affiliations:** 1 Department of Internal Medicine, College of Medicine, Seoul National University, Daehak-ro 101, Jongno-gu, Seoul, 03080 Republic of Korea; 2 Cardiovascular Center, Seoul National University Hospital, Seoul, Republic of Korea; 3 Department of Cardiology, Cardiovascular Center, Bundang Hospital, Seoul National University, Gumiro 166, Bundang, Seongnam, Gyeonggi-do Republic of Korea; 4 Department of Cardiology in Internal Medicine, Chungnam National University Hospital, Chungnam National University College of Medicine, Daejeon, Republic of Korea

**Keywords:** Cardiology, Cardiovascular diseases

## Abstract

We aimed to investigate the sex differences in associations of diabetes mellitus (DM) with echocardiographic phenotypes and clinical outcomes of heart failure (HF). We studied 4,180 patients admitted for acute HF between 2009 and 2016 (median follow-up, 31.7 months) whose left ventricular global longitudinal strain (LV-GLS) data were available. Patients were compared by sex and DM. Structural equation model (SEM) analysis was performed to evaluate the moderating effects of two causal paths, via ischemic heart disease (IHD) and LV-GLS, linking DM with mortality. Compared to non-diabetic women, diabetic women had significantly lower LV-GLS (11.3% versus 10.1%, p < 0.001), but the difference was attenuated within men (9.7% versus 9.2%, p = 0.014) (p-for-interaction by sex = 0.018). In Cox analyses, DM was an independent predictor for higher mortality in both sexes (women: adjusted hazard ratio [HR] 1.35, 95% confidence interval [CI] 1.15–1.59 versus men: HR 1.24, 95% CI 1.07–1.44, p-for-interaction by sex = 0.699). Restricted cubic spline curves showed that LV-GLS consistently declined, and mortality increased in women with worsening hyperglycemia, but these trends were not evident in men. In SEM analysis, the main driver from DM to mortality differed by sex; men had a stronger effect via IHD than LV-GLS, whereas LV-GLS was the only predominant path in women.

## Introduction

Despite the advances in the management of heart failure (HF), re-hospitalization and mortality rates remain distressingly high^1^. The need for further understanding of the pathophysiology of HF is thus imperative, and efforts to improve the risk stratification of HF patients based on underlying pathophysiology are ongoing. Although the pathophysiology of HF includes diverse mechanisms, neurohormonal disturbances and oxidative stress have been recognized as major contributing factors^2^. Intriguingly, these mechanisms are also important for the development of diabetes mellitus (DM)^3^. Indeed, DM is highly prevalent in HF, accounting for up to 40% of HF patients^4^, and also associated with a worse prognosis^5^. These findings suggest the importance of detection and management of DM in individuals at risk for or with HF.

On the other hand, accumulating evidence proposes that there are significant sex differences in the cardiovascular consequences of DM, including the development of HF^6^. A recent meta-analysis demonstrated that the excess risk of HF associated with DM was significantly higher in women than in men^7^. Considering that HF in women occurred with less ischemic etiology than in men^8^, and ischemic heart disease (IHD) in diabetic patients had an especially deleterious impact on mortality^9^, it can be assumed that the prognosis of women with HF and DM might differ from men. However, there is a relative paucity of data on sex differences in the association of DM with clinical outcomes of HF, although most registries suggest that women with HF survive better than men with HF^10^.

We hypothesized that the impact of DM on clinical and echocardiographic characteristics of patients with HF and their prognosis differs by sex. This study aimed to investigate the association of DM with left ventricular (LV) systolic function, measured as LV global longitudinal strain (LV-GLS), and mortality according to sex in HF patients, from the cohort of patients with acute HF.

## Results

**Figure 1:**
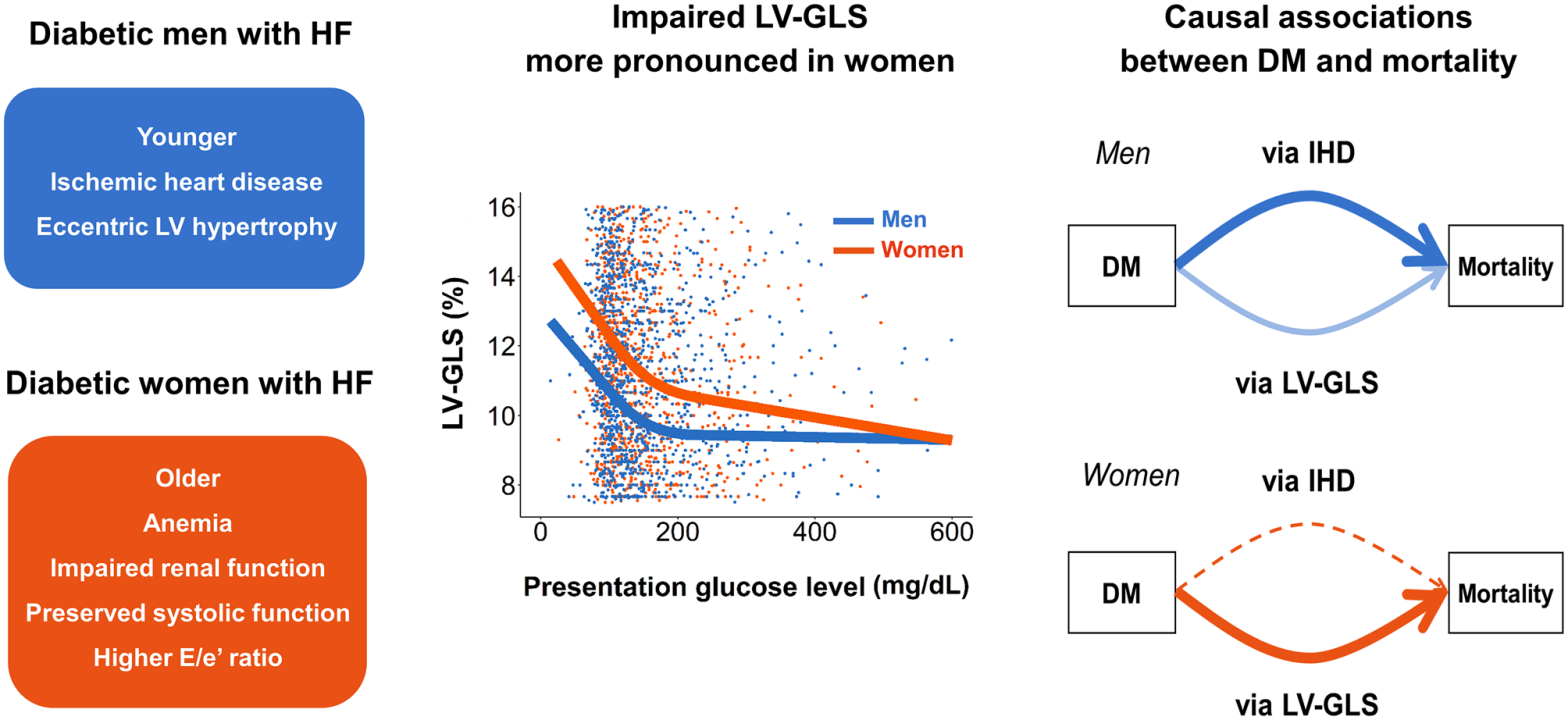
Sex-related differences in the impact of DM on phenotypes, LV-GLS, and causal associations in HF. The main findings of the study are summarized. (*Left*) Diabetic men and women with HF had different clinical and echocardiographic phenotypes. (*Middle*) The associations of presentation blood glucose level with the LV-GLS impairment were more pronounced in women. In the RCS curves, LV-GLS continually declined as hyperglycemia became severe in women, while it reached a plateau in men, resulting in the gradual convergence of the two curves. (*Right*) The main driver from DM to mortality differed; men had a larger effect via IHD than LV-GLS impairment, whereas effect mediating LV-GLS was the only predominant path in women. Dominant pathways are indicated by bold arrows; those with dashes arrows are statistically insignificant. DM = diabetes mellitus; HF = heart failure; IHD = ischemic heart disease; LV-GLS = left ventricular global longitudinal strain; RCS = restricted cubic spline.

The main findings is summarized in Fig. 1.

### Clinical and echocardiographic features by DM

Among 4,180 patients with HF (mean 70.7 years), 1,431 (34.2%) had DM, with more prevalence in men than women (792 [35.7%] versus 639 [32.6%], p = 0.036) (Supplementary Table S1). Baseline characteristics according to sex and DM are summarized in Table 1. In both men and women, diabetic patients had higher body mass index, and more prevalent hypertension and IHD compared to non-diabetic patients (Table 1). DM was also significantly associated with anemia, lower sodium level, and impaired renal function in both sex. Regarding the echocardiographic parameters, women had higher LV ejection fraction (LV-EF) and LV-GLS, smaller LV dimensions, and more frequent concentric LV hypertrophy (LVH) than men (Supplementary Table S1). When comparing echocardiography parameters according to sex and DM, diabetic men and women had a higher E/e’ ratio and more concentric LVH compared to non-diabetic counterparts (Table 2).

**Table 1:** Baseline characteristics of the study participants according to sex and DM status.

	Men			Women		
	Non-DM (n = 1426)	DM (n = 792)	P-value	Non-DM (N = 1323)	DM (N = 639)	P-value
Age, year	70.0 (58.0–78.0)	71.0 (62.0–77.0)	0.153	77.0 (68.0–83.0)	75.0 (69.0–81.0)	0.126
BMI, kg/m^2^	23.0 (20.6–25.5)	23.7 (21.5–25.9)	< 0.001	22.4 (19.8–25.3)	23.7 (21.3–26.7)	< 0.001
SBP, mmHg	122 (108–140)	127 (110–146)	0.002	125 (110–142)	130 (111–151)	0.001
DBP, mmHg	72 (62–83)	71 (62–83)	0.313	71 (62–82)	72 (62–82)	0.767
Heart rate, bpm	85 (70–103)	85 (72–101)	0.695	85 (70–103)	87 (71–102)	0.319
NYHA class, n (%)			0.165			0.017
I/II	82 (9.0)	50 (8.4)		79 (9.0)	23 (5.0)	
III	514 (56.4)	310 (52.2)		480 (54.6)	249 (53.9)	
IV	315 (34.6)	234 (39.4)		320 (36.4)	190 (41.1)	
Past medical history, n (%)						
Hypertension	626 (43.9)	574 (72.5)	< 0.001	714 (54.0)	481 (75.3)	< 0.001
IHD	409 (28.7)	396 (50.0)	< 0.001	289 (21.8)	260 (40.7)	< 0.001
Atrial fibrillation	458 (32.1)	173 (21.8)	< 0.001	449 (33.9)	150 (23.5)	< 0.001
Laboratory findings						
TC, mg/dL	147 (120–176)	144 (117–173)	0.046	155 (131–188)	152 (123–185)	0.018
Hemoglobin, g/L	13.3 (11.3–14.7)	12.5 (10.6–14.0)	< 0.001	11.9 (10.4–13.2)	11.0 (9.8–12.4)	< 0.001
Sodium, mmol/L	138 (135–140)	137 (134–139)	< 0.001	138 (134–140)	136 (133–139)	< 0.001
Potassium, mmol/L	4.1 (3.8–4.5)	4.3 (3.8–4.7)	< 0.001	4.0 (3.7–4.5)	4.3 (3.8–4.8)	< 0.001
Troponin I, ng/mL	0.1 (0.0–1.3)	0.2 (0.0–2.8)	< 0.001	0.1 (0.0–0.6)	0.1 (0.0–1.5)	0.001
AST, IU/L	29.0 (20.0–45.0)	25.0 (18.0–39.0)	< 0.001	27.0 (19.0–41.0)	24.0 (17.0–35.5)	< 0.001
ALT, IU/L	23.0 (14.0–39.0)	20.0 (12.0–35.0)	< 0.001	18.0 (11.0–32.0)	17.0 (11.0–27.0)	0.019
BUN, mg/dL	21.0 (15.4–29.0)	24.0 (17.0–36.0)	< 0.001	19.0 (15.0–28.0)	22.6 (17.0–34.3)	< 0.001
Creatinine, mg/dL	1.1 (0.9–1.5)	1.3 (1.0–2.1)	< 0.001	0.9 (0.7–1.2)	1.1 (0.8–1.7)	< 0.001
GFR, mL/min/1.73m^2^	67.5 (45.7–85.8)	52.9 (30.1–77.1)	< 0.001	64.7 (42.7–84.2)	48.4 (28.6–72.9)	< 0.001
HbA1c, %*	5.8 (5.5–6.1)	7.0 (6.5–8.0)	< 0.001	5.8 (5.5–6.1)	7.0 (6.5–8.1)	< 0.001
Presentation glucose level, mg/dL	116 (99–142)	169 (125–234)	< 0.001	118 (100–146)	172 (127–244)	< 0.001
NT-proBNP, pg/mL	4014 (1481–8745)	5008 (2090–13,870)	< 0.001	4799 (1845–11,735)	5253 (1752–13,874)	0.285
Medication, n (%)						
Beta-blockers	830 (58.2)	519 (65.5)	0.010	774 (58.5)	428 (67.0)	0.002
RAS-blockers	977 (68.5)	566 (71.5)	0.670	859 (64.9)	455 (71.2)	0.025
Spironolactone	640 (44.9)	338 (42.7)	0.124	617 (46.6)	282 (44.1)	0.187
Diuretics	988 (69.3)	580 (73.2)	0.534	990 (74.8)	485 (75.9)	0.651
Statins	668 (46.8)	543 (68.6)	< 0.001	607 (45.9)	417 (65.3)	< 0.001

Values given as number (percentage), or median (interquartile range) unless otherwise indicated.

*HbA1c data was available in 42.3% patients.

ALT = alanine aminotransferase; AST = aspartate aminotransferase; BMI = body mass index; BUN = blood urea nitrogen; DBP = diastolic blood pressure; DM = diabetes mellitus; GFR = glomerular filtration rate; HbA1c = glycated hemoglobin; IHD = ischemic heart disease; NT-proBNP = N-terminal pro-brain natriuretic peptide; NYHA = New York Heart Association; RAS = renin-angiotensin system; SBP = systolic blood pressure; TC = total cholesterol.

**Table 2:** Echocardiography characteristics of the study participants according to sex and DM status.

	Men			Women		
	Non-DM (n = 1,426)	DM (n = 792)	P-value	Non-DM (N = 1,323)	DM (N = 639)	P-value
LVEDD, mm	56.0 (50.0–63.0)	55.0 (50.0–61.0)	0.038	50.0 (44.2–56.0)	50.0 (45.0–55.0)	0.921
LVESD, mm	44.0 (36.0–52.9)	43.8 (36.0–52.0)	0.254	36.0 (29.0–44.0)	36.0 (29.0–45.0)	0.599
LVEDV, mL	130 (97–180)	129 (93–170)	0.019	86 (62–120)	91 (65–125)	0.058
LVESV, mL	85 (54–130)	84 (49–123)	0.088	48.0 (27.9–79.8)	51.6 (31.7–85.1)	0.028
LV-EF, %	34.7 (25.0–49.0)	34.0 (25.0–48.4)	0.554	45.0 (32.0–58.0)	42.1 (30.0–57.0)	0.003
HFpEF, n (%)	337 (23.6)	177 (22.3)	0.526	557 (42.1)	226 (35.4)	0.005
LA diameter, mm	45.0 (39.0–52.0)	44.5 (39.7–50.7)	0.251	44.0 (38.0–50.0)	43.0 (38.5–48.6)	0.309
LA volume, mL	86 (62–119)	81 (63–111)	0.040	84 (61–119)	78 (59–100)	0.001
LAVI, mL/m^2^	50.3 (36.3–69.0)	46.7 (36.6–62.3)	0.014	57.0 (40.0–80.7)	50.4 (38.7–65.7)	< 0.001
E wave, m/s	0.8 (0.6–1.0)	0.8 (0.6–1.1)	0.017	0.9 (0.6–1.1)	0.9 (0.6–1.2)	0.051
A wave, m/s	0.6 (0.5–0.8)	0.7 (0.5–0.9)	< 0.001	0.8 (0.6–1.0)	0.9 (0.7–1.1)	0.006
Deceleration time, s	160 (125–206)	156 (124–198)	0.203	170 (135–227)	168 (133–226)	0.488
E/e’ ratio	15.0 (10.5–21.4)	17.4 (12.3–24.3)	< 0.001	16.7 (11.8–22.9)	18.8 (14.5–25.9)	< 0.001
Septum, mm	10.0 (9.0–12.0)	10.7 (9.1–12.0)	0.052	10.0 (9.0–11.0)	10.0 (9.0–11.8)	0.001
Posterior wall, mm	10.0 (9.0–11.4)	10.0 (9.0–11.5)	0.699	10.0 (9.0–11.0)	10.0 (9.0–11.0)	0.001
LVMI, g/m^2^	133 (107–164)	133 (107–157)	0.096	123 (98–150)	123 (103–148)	0.632
RWT	0.4 (0.3–0.4)	0.4 (0.3–0.5)	0.077	0.4 (0.3–0.5)	0.4 (0.3–0.4)	0.050
LVH, n (%)	840 (58.9)	468 (59.1)	0.990	910 (68.8)	469 (73.4)	0.017
LVH type, n (%)			0.078			0.058
Concentric LVH	236 (28.1)	154 (32.9)		354 (38.9)	208 (44.3)	
Eccentric LVH	604 (71.9)	314 (67.1)		556 (61.1)	261 (55.7)	
LV-GLS, %	9.7 (6.5–13.8)	9.2 (6.3–12.6)	0.014	11.3 (8.1–15.4)	10.1 (7.0–14.1)	< 0.001
RV-FAC, %	36.8 (24.0–46.3)	39.0 (26.0–50.0)	0.013	39.2 (27.0–49.6)	39.3 (29.3–50.2)	0.375

Values given as number (percentage), or median (interquartile range) unless otherwise indicated.

HFpEF, heart failure with preserved ejection fraction; LA, left atrium; LAVI, left atrium volume index; LV, left ventricle; LVEDD, LV end-diastolic diameter; LVEDV, LV end-diastolic volume; LV-EF, LV ejection fraction; LVESD, LV end-systolic diameter; LVESV, LV end-systolic volume; LV-GLS, LV global longitudinal strain; LVH, LV hypertrophy; LVMI, LV mass index; RV-FAC, right ventricular fractional area change; RWT, relative wall thickness.

### Sex difference in the association of DM with clinical and echocardiographic features

DM severity assessed by glycosylated hemoglobin (HbA1c) and presentation glucose levels was similar between men and women (Supplementary Table S1); however, clinical and echocardiographic features significantly differed when stratified by sex and DM. Compared to diabetic men, diabetic women were older and had lower hemoglobin level and glomerular filtration rate, whereas diabetic men more often had IHD with elevated troponin I level (Table 1). DM was associated with more frequent LVH in women, but the difference was not identified among men. Diabetic women had the highest proportion of concentric LVH among the four groups (44.3% of LVH), as well as the highest E/e’ ratio (18.8 [IQR, 14.5–25.9]) (Table 2). Of note, diabetic women had significantly lower LV-EF than non-diabetic women (42.1% [IQR, 30.0–57.0%] vs. 45.0% [IQR, 32.0–58.0%], p = 0.003), whereas no significant difference was observed between diabetic and non-diabetic men (34.0% [IQR, 25.0–48.4%] vs. 34.7% [IQR, 25.0–49.0%], p = 0.554) (p-for-interaction by sex = 0.019). In both sex, however, LV-GLS was significantly lower in diabetic patients than non-diabetics, with a more prominent difference in women (10.1% [IQR, 7.0–14.1%] vs. 11.3% [IQR, 8.1–15.4%], p < 0.001 for women; 9.2% [IQR, 6.3–12.6%] vs. 9.7% [IQR, 6.5–13.8%], p = 0.014 for men) (p-for-interaction by sex = 0.018).

### Mortality risk according to sex and DM

**Figure 2:**
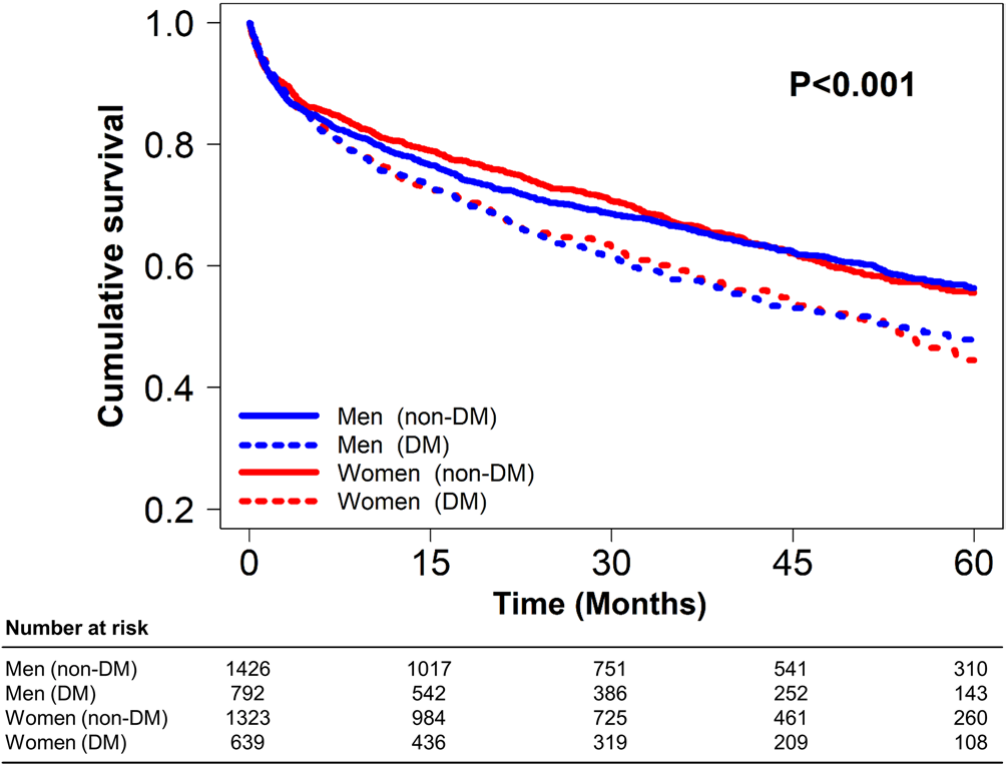
Kaplan–Meier curves for 5-year all-cause mortality according to sex and DM.

During a median of 31.7 months (IQR, 11.6–54.3 months), 1,765 deaths occurred. 5-year mortality according to sex and DM is shown (Fig. 2). Among the four groups, non-diabetic women had the lowest mortality during the early follow-up period, which was non-significantly lower than that of non-diabetic men. The difference in mortality rates between non-diabetic women and non-diabetic men gradually decreased and became similar at a longer follow-up of 5 years (p = 0.773). The mortality rates between diabetic women and diabetic men were consistently similar throughout the entire follow-up.

Cox analysis showed that DM was significantly associated with increased unadjusted and adjusted risks of death in both sex (Table 3). The magnitude of hazard ratio for mortality between DM and non-DM was greater in women than men, although not significant (adjusted hazard ratio = 1.35 [95% confidence interval: 1.15–1.59] versus 1.24 [1.07–1.44], p-for-interaction = 0.699) (Table 3).

**Table 3:** Association of diabetes mellitus with the 5-year mortality in men and women with heart failure.

Outcome	Men				Women			
	Unadjusted HR (95% CI)	P-value	Adjusted HR (95% CI)	P-value	Unadjusted HR (95% CI)	P-value	P-value (95% CI)	Adjusted HR
DM	1.27 (1.11–1.45)	< 0.001	1.24 (1.07–1.44)	0.005	1.32 (1.15–1.53)	< 0.001	1.35 (1.15–1.59)	< 0.001
Non-DM	1.00 (reference)	–	1.00 (reference)	–	1.00 (reference)	–	1.00 (reference)	–

CI = confidence interval; DM = diabetes mellitus; HR = hazard ratio.

When patients were stratified according to the tertile values of HbA1c (< 5.7%, 5.7–7.0%, > 7.0%), there was a stepwise increase in mortality with worsening HbA1c levels in women (p = 0.026), but it was not significant among men (p = 0.133) (Supplementary Figure S1). While men with mid-range HbA1c levels (5.7–7.0%) had a long-term outcome similar to those with lower HbA1c levels (< 5.7%), the survival of women with mid-range HbA1c levels was approaching that of women with the highest HbA1c levels (> 7%) (Supplementary Figure S1).

### Sex-specific associations of presentation glucose level with mortality

**Figure 3:**
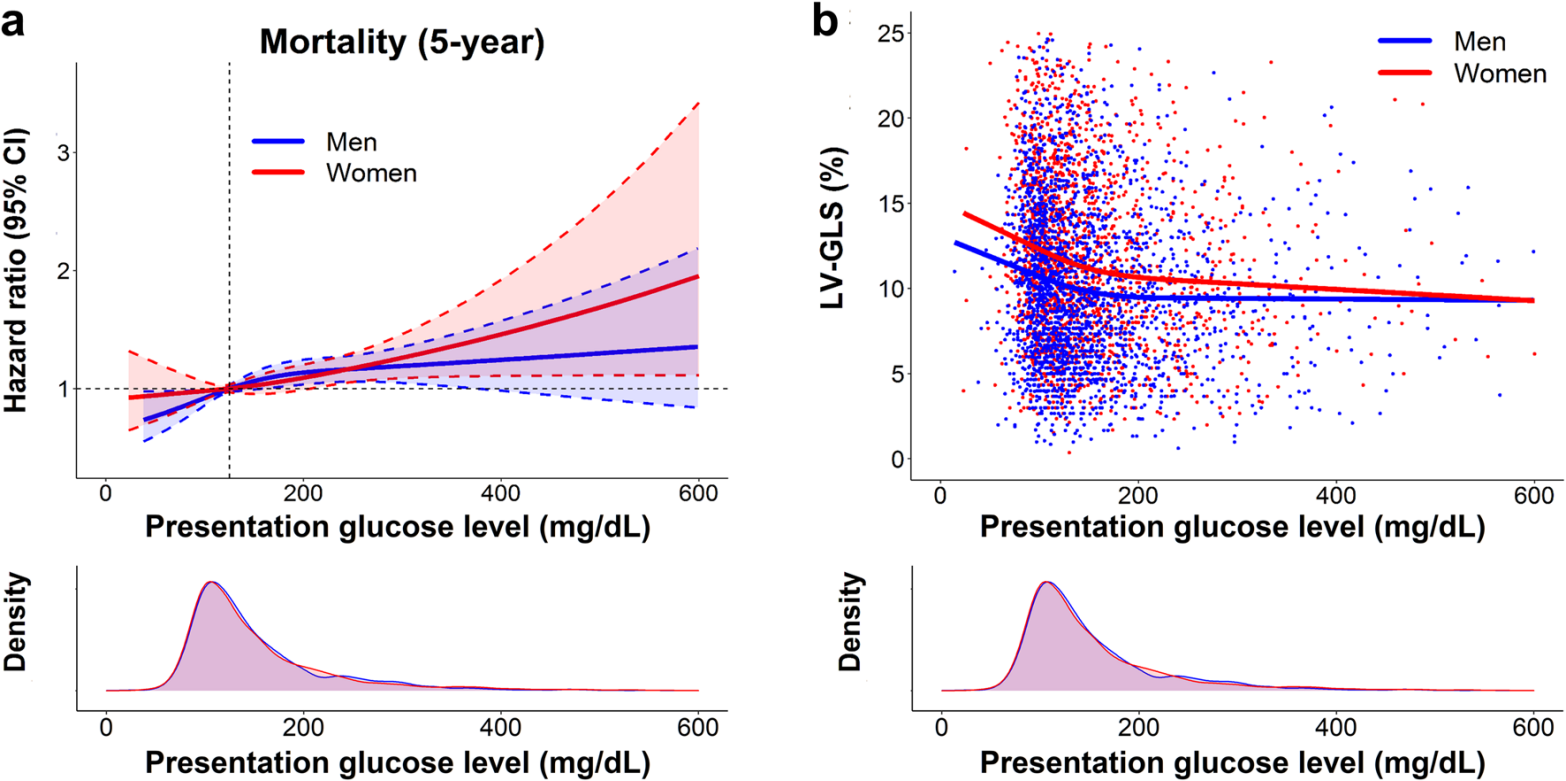
Sex-specific association of presentation glucose level with mortality risk and LV-GLS. (**a**) The graph shows the adjusted hazard ratios (solid lines) and 95% confidence intervals (dashed lines and shaded area) for the association between presentation glucose level and 5-year mortality in men (blue) and women (red). The glucose level was modeled with RCS in Cox models. The reference of glucose level was set at 125 mg/dL for hazard ratios estimation. Density plots show the distribution of patients according to the glucose level. (**b**) RCS curves were plotted between presentation glucose level and LV-GLS. Each dot indicates an individual patient’s data. LV-GLS = left ventricular global longitudinal strain, RCS = restricted cubic spline.

The restricted cubic spline (RCS) curves showed the associations of presentation blood glucose level with 5-year mortality risk according to sex (Fig. 3a). Data on presentation glucose level was available in 4125 (98.7%), consisting of 2183 men and 1942 women, and all these patients were included in the RCS analysis irrespective of DM status. The patterns of RCS curves differed by sex. At the 5-year follow-up, the risk of mortality continually increased as glucose level rise in women, while the linear increase pattern was less pronounced in men, particularly if glucose level exceeds 200 mg/dL (Fig. 3a). Density plots showed that the distribution of patients according to glucose level was similar between sex.

### Sex-specific associations of presentation glucose level with LV-GLS

The associations between presentation glucose level and LV-GLS in men and women are examined. In the simple linear regression model, there was a negative correlation between presentation glucose level and LV-GLS, with a stronger association in women (r = − 0.10 in men, p < 0.001, r = − 0.14 in women, p < 0.001, p-for-interaction by sex = 0.058). The RCS curves demonstrated a marked nonlinear relationship between presentation glucose level and LV-GLS by sex (Fig. 3b). Overall, the gradual decrease in LV-GLS was observed with an increase in the glucose level, approximately until 200 mg/dL in both sex (Fig. 3b). When the glucose level exceeded 200 mg/dL, LV-GLS further declined approximately from 12 to 10% in a dose-dependent manner in women. In men, however, LV-GLS decreased to around 10% at the glucose level of 200 mg/dL and reached a plateau thereafter, resulting in the gradual convergence of the two curves (Fig. 3b).

Regarding the effect of hyperglycemia on diastolic function, E/e’ ratio increased as the presentation blood glucose level increased up to approximately 200 mg/dL and reached a plateau thereafter in both sexes. Women had a higher E/e’ ratio than men for the same degree of hyperglycemia (Supplementary Figure S2).

### Sex differences in regression paths between DM and mortality

**Figure 4:**
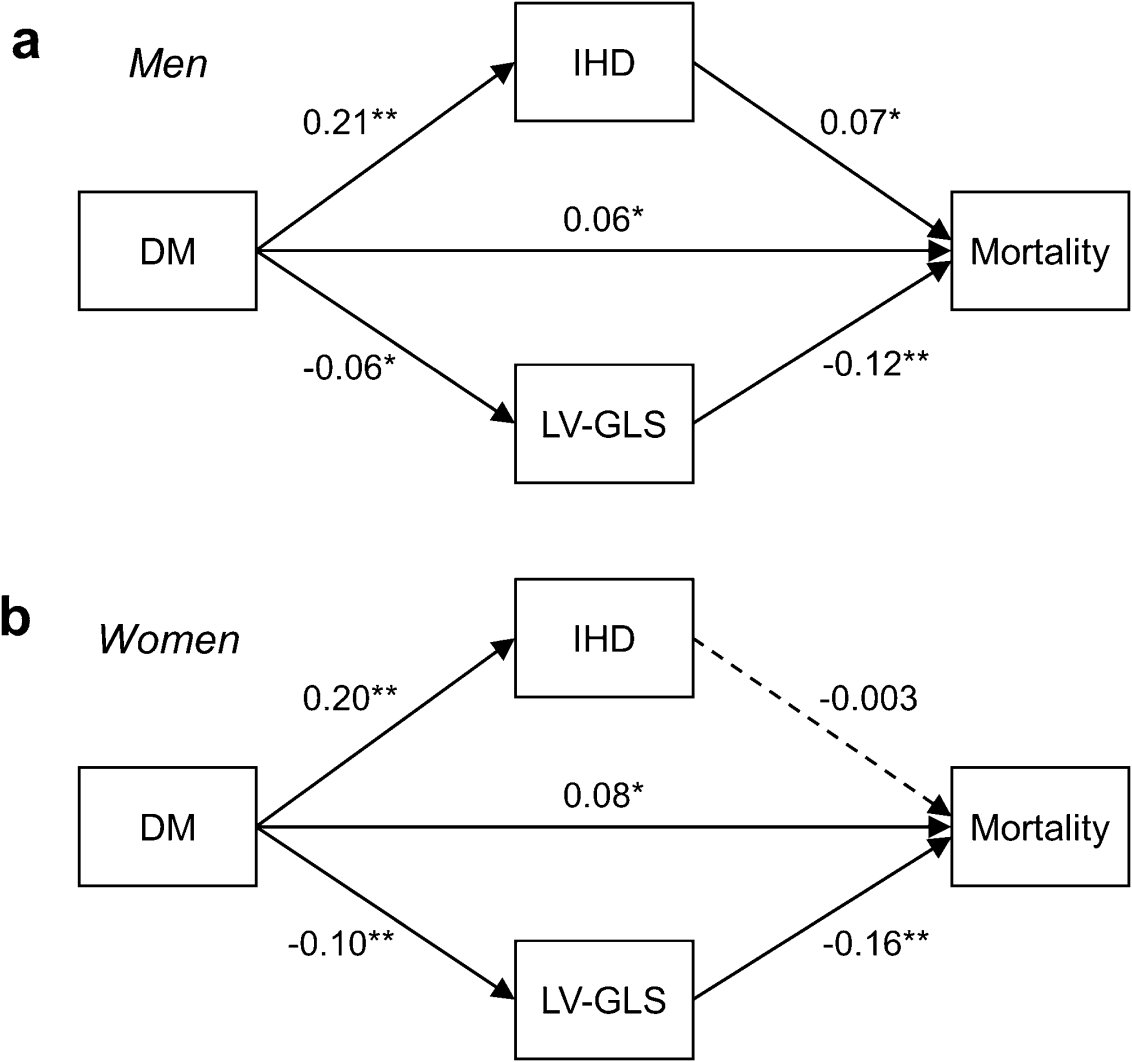
Path diagrams of relationship between DM, LV-GLS or IHD, and mortality by sex. Diagrams of the structural equation model in men (**a**) and women (**b**). Standardized path coefficients are shown on each path as effect estimates. Solid lines denote significant paths and dashed lines, non-significant paths. 5-year mortality data was used. DM = diabetes mellitus; IHD = ischemic heart disease; LV-GLS = left ventricular global longitudinal strain. ^*^p < 0.05, ^**^p < 0.001.

The structural equation model (SEM) diagrams with standardized path coefficients are presented for each sex (Fig. 4). This model included a direct path from DM to mortality, with two indirect paths from DM to mortality via IHD and LV-GLS as intermediate mediators, and it had an adequate statistical fit (Supplementary Table S2). The direct path from DM to mortality was significant in both men and women. Regarding the indirect paths, the path from DM to LV-GLS was significant in both sex, with a larger coefficient for women (coefficient = − 0.10, p < 0.001) than men (coefficient = − 0.06, p = 0.004). The path from DM to IHD was also significant in both sex. However, the path from IHD to mortality was significant in men (coefficient = 0.07, p = 0.001), but not in women (coefficient = − 0.003, p = 0.890), while the path from LV-GLS to mortality was significant in both sex.

Table 4 summarizes the standardized coefficients of direct and indirect effects. In men, the indirect effect mediated through IHD was greater (DM–IHD–mortality path: coefficient = 0.015, p = 0.001) than that mediated through LV-GLS (DM–LV-GLS–mortality path: coefficient = 0.008, p = 0.009). In women, however, the indirect effect via IHD was markedly smaller than that in men, and not significant (DM–IHD–mortality path: coefficient = − 0.001, p = 0.890). The indirect effect mediated through LV-GLS was significant and more pronounced in women (DM–LV-GLS–mortality path: coefficient = 0.015, p < 0.001) compared to men. Similar findings were observed in the sensitivity analysis using the presentation glucose or HbA1c level instead of DM (Supplementary Figure S3 and Supplementary Table S3).

**Table 4:** Coefficients and standard errors of the structural equation path models of direct and indirect effects of DM for mortality.

Causal paths			Total effects	Direct effects	Indirect effects	Standard Error	P-value
Men			0.078			0.021	< 0.001
DM		→ Mortality		0.055		0.021	0.011
DM	→ IHD	→ Mortality			0.015	0.005	0.001
DM	→ LV-GLS	→ Mortality			0.008	0.003	0.009
Women			0.090			0.022	< 0.001
DM		→ Mortality		0.076		0.023	0.001
DM	→ IHD	→ Mortality			− 0.001	0.004	0.890
DM	→ LV-GLS	→ Mortality			0.015	0.004	< 0.001

SEM models were constructed to identify the direct and indirect effects of DM on the mortality. 5-year death data was used for mortality.

DM = diabetes mellitus; IHD = ischemic heart disease; LV-GLS = left ventricular global longitudinal strain; SEM = structural equation modeling.

When we repeated analyses after incorporating the E/e’ ratio as a third intermediate mediator, the results were materially unchanged. Specifically, in men, IHD had the largest mediating effect, but the mediating effect via E/e’ ratio or LV-GLS was not statistically significant. In women, the E/e’ ratio and LV-GLS had the largest and similar mediating effects, but the mediating effect via IHD was not statistically significant (Supplementary Figure S4 and Supplementary Table S4).

## Discussion

The main findings of our study can be summarized as follows: (1) clinical and echocardiographic features of patients with acute HF significantly differed by sex and DM, (2) DM was a significant and independent predictor for increased mortality in both men and women, (3) although the effect of DM on mortality was similarly significant in both sexes, the predominant mediating factor between DM and mortality was different, namely, LV-GLS for women and IHD for men, and 4) the associations of presentation blood glucose level with LV-GLS impairment were more pronounced in women than men (Fig. 1).

Multiple evidence support that there are significant sex differences in the characteristics and prognosis of HF patients. It has been reported that women with HF are more likely to be older, obese, have reduced renal function, and higher LV-EF, whereas men with HF more commonly have IHD^11^. Sex-related differences in DM have also gained intense attention. For instance, women are over-represented as diabetic cardiomyopathy, in contrast to ischemic cardiomyopathy, which is more prevalent in men^12^. However, these sex differences have been evaluated separately for HF and DM. Considering up to 40% of HF patients have DM and 12% of diabetic patients have HF^4^, it is important to consider the integrated effects of HF and DM on sex differences. Our study demonstrated that the association between presentation glucose level and LV-GLS was more prominent in women than men with HF, suggesting a more deleterious impact of hyperglycemia on prognosis in women with HF.

It is worth mentioning that there were significant sex differences in the LV remodeling patterns related to DM in HF patients. When stratifying HF patients into four groups by sex and diabetes status, diabetic women with HF more frequently had LVH compared to the non-diabetic counterpart (p = 0.017), which was not observed among men (p = 0.990) (Table 2). In addition, concentric LVH was most prevalent (44.3%), and E/e’ ratio was highest (18.8 [IQR, 14.5–25.9]) in diabetic women (Table 2), suggesting a female preponderance in the prevalence of HF with preserved EF coexisting with DM. These findings also corroborate with the previous studies highlighting the sex-specific pattern of cardiac remodeling and diastolic function in DM^13,14^. Of note, these cardiac structural and functional abnormalities had a more deleterious impact on prognosis in women than men^14^. Therefore, our study adds support to the concept that a sex-specific approach is key for investigating the pattern of cardiac remodeling and its association with outcomes in HF patients. Furthermore, given recent promising results of the clinical trial showing that dapagliflozin treatment reduced LV mass in diabetic patients with LVH^15^, optimized drug therapy can induce reverse cardiac remodeling, which may lead to improved cardiovascular outcomes in patients with HF and DM, particularly in women.

Mechanisms underlying sex differences in HF are poorly understood. One possible mechanism is the cardio-protective role of sex-hormone in women. Several studies suggest that estrogen protects the heart from various types of stress, including hypertrophic, ischemic, and cytotoxic stimuli^16,17^. On the other hand, menopause, a physiological estrogen withdrawal, was significantly associated with impaired LV systolic performance and concentric LV geometry^18^. Regarding DM, estrogen also exerts various positive effects including insulin sensitivity, protection of pancreatic beta-cell, reduction in hepatic gluconeogenesis, and increase in muscle glucose transporter^19^. These findings imply that estrogen withdrawal from menopause may negatively impact the myocardial function and structure. Our study showed that the detrimental association between glucose level and LV-GLS was more remarkable in women than men, which raises the speculation that adverse effects of DM are possibly accentuated by estrogen withdrawal in women. However, since the data on estrogen levels or menopausal status were unavailable in our study, further studies are needed to test this hypothesis.

Sex-differences in cardiac steatosis can be another possible mechanism for the finding that LV systolic function is more vulnerable to hyperglycemia in women. Cardiac steatosis is characterized by the accumulation of triglyceride into the myocardium in patients with metabolic abnormalities, especially type 2 diabetes^20^. A major consequence of cardiac steatosis is the structural and functional change of heart, including impaired LV myocardial strain^21^. Notably, cardiac steatosis has been reported to be more pronounced in women than men^22^. In our study, LV systolic dysfunction by hyperglycemia was more prominent and consistent in women, which implies that such mechanism may play a role. However, since our study did not have data on cardiac steatosis, this explanation needs further research.

Worse prognosis in diabetic women with HF has been repeatedly reported^13,23^. This finding might stem from the fact that diabetic women have more prevalent comorbidities compared to diabetic men^13^, or that women with HF receive less optimal management^23^. Our findings suggest another possibility that a more pronounced impairment of LV systolic function under hyperglycemia in women could be one biological basis for the female vulnerability. This theory is further supported by our SEM analysis, which showed that LV-GLS was a major moderator between DM and mortality in women, while IHD was a dominant one in men. Hence, our study highlights the importance of sex-specific strategies to improve the prognosis of patients with both HF and DM; more intensive monitoring of the change in LV systolic function is recommended for women while timely detection of concomitant IHD is crucial for men.

### Strengths and limitations

The most compelling advantage of our study is a well-constructed, large imaging database containing LV-GLS from all participants. Additionally, considering the relatively low body mass index in this study population, our findings might be less confounded by overweight or obesity, which is an important confounding factor in the studies investigating the effect of DM.

However, several limitations should be considered when interpreting the results. First, we could not find that these sex differences are translated into significant differences in hard outcomes, probably due to the lack of long-term survival data. Future studies with a larger population and longer follow-up are warranted to validate the long-term consequences of sex-specific association of DM with LV systolic function. Second, analyses based on the other metrics of DM severity, such as fasting glucose level, would have provided additional information. We used blood glucose level at the time of HF presentation for analyses, an index known to have a linear association with adverse outcomes both in diabetic and non-diabetic HF^24,25^. As HbA1c measurements were not routinely performed, it was only available in (42.3%) in our study, which had a moderate correlation with the presentation glucose level in both sex (men: r = 0.55, p < 0.001, women: r = 0.45, p < 0.001) (p-for-interaction by sex = 0.509) (Supplementary Figure S5). Lastly, as Korean patients were exclusively enrolled, it is uncertain whether these results may be generalized to other ethnicities.

## Conclusions

Although diabetic patients with HF had higher mortality than non-diabetic counterparts in both men and women, sex differences were found in clinical and echocardiographic features, and notably, the effect of hyperglycemia on LV-GLS and mortality, with more pronounced associations in women. Furthermore, the major factor intermediating between DM and mortality differed by sex, namely, LV-GLS for women and IHD for men. Our study provides support for the importance of sex-specific strategies for HF management.

## Methods

### Study population

This study utilized data from The STrain for Risk Assessment and Therapeutic Strategies in patients with Acute Heart Failure registry, whose protocol has been previously described^26^. Briefly, 4312 patients admitted to the hospital from HF were prospectively enrolled from 3 tertiary university hospitals between January 2009 and December 2016. Eligible criteria were symptoms and signs compatible with HF, and one of the following: (1) evidence of pulmonary edema on physical examination or chest radiography or (2) objective findings of LV dysfunction or structural heart disease. The lack of LV-GLS data was the main exclusion criterion; echocardiography was performed in 4237 (98.2%), and LV-GLS was measured in 4180 (96.9%), which was the final study sample.

All study protocols were approved by the ethics committees at each center (Seoul National University Hospital, Seoul National University Bundang Hospital, Chungnam National University Hospital), and conformed with the Declaration of Helsinki. As anonymized and unidentified information was used for the analysis, the need for written informed consent was waived by the same ethics committees at each center.

### Variables and definitions

Data on demographics, medical history, and laboratory tests were collected by each center. We defined DM as a chart-documented diagnosis of DM and/or treatment with glucose-lowering medications. Presentation glucose level, which was defined as the initial serum glucose level measured at the time of HF admission^24,25^, was collected irrespective of DM status, and the HbA1c level was obtained within a 1-month period. We defined IHD as one of the following: a history of myocardial infarction or coronary revascularization, or significant coronary stenosis (> 50% epicardial artery stenosis on coronary angiography or computed tomography angiography) or ischemia (perfusion defect on myocardial single photon emission computed tomography).

Patients were categorized into 4 groups by sex and DM: diabetic men, non-diabetic men, diabetic women, and non-diabetic women. The primary outcome was 5-year all-cause mortality. Patients’ vital statuses were obtained from the national insurance data or national death records.

### Echocardiography and strain analysis

Echocardiography was performed following contemporary guidelines^27^, and the details are described in Supplementary Methods. The median time interval between HF admission and echocardiography was 1 day (IQR, 0–2 days).

Echocardiography images were subsequently analyzed for strain measurement at the strain core laboratory. Briefly, images qualified for the strain analysis were uploaded to TomTec software (Image Arena 4.6, Munich, Germany) for deformation analysis. Speckles were automatically tracked frame by frame, aligning to the endocardial border of the myocardium, and LV-GLS was calculated as the averaged values from 3 apical views of the entire LV. All strain measurements were performed by independent observers blinded to participants’ clinical information. We used the absolute value of LV-GLS for a straightforward interpretation.

### Statistical analysis

Categorical variables are presented as frequencies (percentages), and continuous variables as median (interquartile range). The difference between groups was compared using the χ^2^ test or Fisher’s exact test for categorical variables, and Student’s *t-*test or Wilcoxon’s rank-sum test for continuous variables. Analyses investigating the interactions between sex and each continuous variable were also performed using two-way analysis of variance. The cumulative survival was plotted and compared using Kaplan–Meier curves and log-rank test. Cox proportional hazard analyses were performed to evaluate the association between DM and mortality, expressed as hazard ratios with 95% confidence intervals. Multivariate Cox analyses were adjusted for the variables with p < 0.05 in univariable analysis and known risk factors in HF^28^, of which the data is available with missing values < 10%, without multicollinearity: age, body mass index, hypertension, IHD, sodium level, glomerular filtration rate, LV-GLS, and use of beta-blocker and renin-angiotensin system blocker.

The RCS curves were plotted to evaluate the relationship between presentation glucose levels and adjusted hazard ratios of mortality, with 3 knots at the 10th, 50th, 90th percentiles of glucose levels (men: 90, 127, and 243 mg/dL, women: 91, 129, and 246 mg/dL). We also used RCS curves for displaying the relationship between presentation glucose levels and LV-GLS.

To further elucidate which intermediate variables lie on a causal path from exposure (i.e., DM) to outcome (i.e., mortality), we performed the SEM analysis, which is a powerful statistical method to assess complex and multivariate relationships by using several regression equations simultaneously, enabling measurement of both direct and indirect effects between variables^29^. In this study, we examined the contribution of two intermediate variables (LV-GLS and IHD) between DM and mortality, where the magnitude of each path was calculated as path coefficients^29^. LV-GLS and IHD were selected as intermediate variables since myocardial contractile dysfunction and ischemia are known to be the major contributors to cardiovascular mortality in HF patients, especially those with DM^9,30^. Separate SEM analyses were performed for each sex based on our hypothesis that there would be sex-difference in the paths connecting DM and mortality. The final models were depicted as diagrams, with standardized path coefficients and P-values. The *lavaan* and *sem* package in R was used^31^. For a more detailed description of the SEM model, see Supplementary methods.

A two-tailed P-value < 0.05 was considered statistically significant. All analyses were performed using R (version 3.6.0, Vienna, Austria).

## Supplementary Information

Supplementary Information.

## Data Availability

The datasets generated during and/or analysed during the current study are available from the corresponding author on reasonable request.

## Abbreviations

DM: Diabetes mellitus
HF: Heart failure
IHD: Ischemic heart disease
LV-EF: Left ventricular ejection fraction
LV-GLS: Left ventricular global longitudinal strain
LVH: Left ventricular hypertrophy
RCS: Restricted cubic splines
SEM: Structural equation model
