# Supplementary material for: Sex-specific impact of diabetes mellitus on left ventricular systolic function and prognosis in heart failure
Source: Sci Rep. 2021 Jun 3;11:11664. doi: 10.1038/s41598-021-91170-x (PMC8175704; doi:10.1038/s41598-021-91170-x)
Supplement: Supplementary file 1 — Supplementary Information. [file 41598_2021_91170_MOESM1_ESM.docx]

**SUPPLEMENTARY INFORMATION**

**Title:** Sex-specific impact of diabetes mellitus on left ventricular systolic function and prognosis in heart failure

**Authors:** Soongu Kwak, MD, In-Chang Hwang MD, Jin Joo Park MD, PhD, Jae-Hyeong Park MD, PhD, Jun-Bean Park, MD, PhD and Goo-Yeong Cho MD, PhD

**Supplementary Methods**

**Echocardiographic exams**

Echocardiography was performed according to contemporary guidelines,^1^ with current echocardiography machines. Briefly, from the standard parasternal view, left ventricular (LV) internal diameters and wall thickness were measured, and LV mass was calculated using the American Society of Echocardiography method.^2^ Simpson’s biplane method was used to calculate LV ejection fraction (LV-EF). Early and late mitral inflow velocities were acquired at the tip level of the mitral valve from the apical 4-chamber view. Tissue Doppler velocities were obtained at the medial side of the mitral annulus. Relative wall thickness (RWT) of the LV was calculated by the equation as follows: (2 × posterior wall thickness)/LV end-diastole diameter. LV hypertrophy (LVH) was defined as LV mass indexed to body surface area greater than 115 g/m² in men, and 95 g/m² in women, and was further categorized as concentric LVH if RWT >0.42 and eccentric LVH if RWT ≤0.42.^1^

**Equations used for the structural equation model and the example of R codes**

**
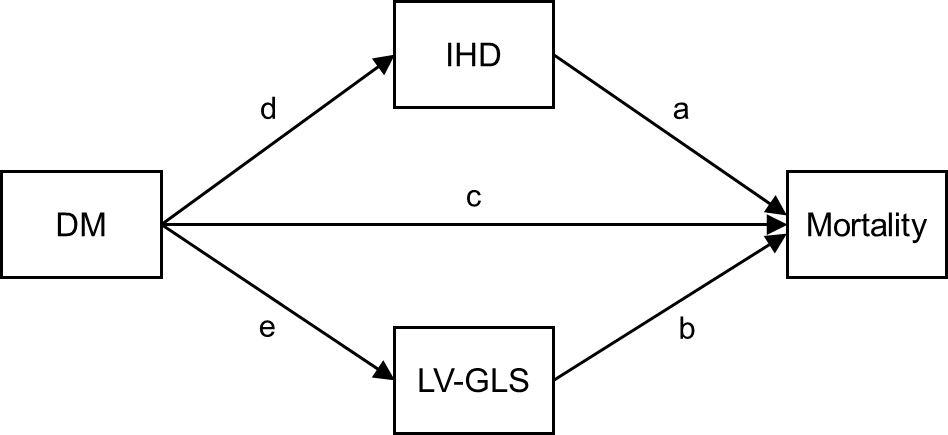
**

Our model consists of one independent variable (DM), two mediator variables (IHD and LV-GLS), one outcome (mortality), one direct path (effect: c), and two indirect paths (effect: a-d and b-e).^3^ To estimate each effect in the diagram, we solved the equations as follows (*sem* library in R).

**Structural equation models (R codes for *sem* library):**

Mortality ~ a* IHD + b* LV-GLS + c* DM

IHD ~ d*DM

LV-GLS ~ e*DM

ad := a*d ## indirect effect mediating IHD

be := b*e ## indirect effect mediating LV-GLS

total := c + a*d + b*e ## total effect from DM to Mortality

**Results (example for the SEM in women, Table 4 and Fig 4b):**

|  | Standardized coefficient | Standard error | Z-value | P-value |
| --- | --- | --- | --- | --- |
| **Regressions:** |  |  |  |  |
| Mortality ~ IHD (a) | -0.003 | 0.023 | -0.138 | 0.890 |
| Mortality ~ LV-GLS (b) | -0.155 | 0.022 | -7.050 | <0.001 |
| Mortality ~ DM (c) | 0.076 | 0.023 | 3.361 | 0.001 |
| IHD ~ DM (d) | 0.197 | 0.021 | 9.153 | <0.001 |
| LV-GLS ~ DM (e) | -0.099 | 0.022 | -4.456 | <0.001 |
| **Defined parameters:** |  |  |  |  |
| ad | -0.001 | 0.004 | -0.138 | 0.890 |
| be | 0.015 | 0.004 | 3.757 | <0.001 |
| total | 0.090 | 0.022 | 4.074 | <0.001 |

**Reference for Supplementary Methods**

1. Lang, R.M., et al. Recommendations for cardiac chamber quantification by echocardiography in adults: an update from the American Society of Echocardiography and the European Association of Cardiovascular Imaging. *J Am Soc Echocardiogr* **28**, 1-39 (2015).

2. Seo, H.Y., et al. Discrepancies in Left Ventricular Mass Calculation Based on Echocardiography and Cardiovascular Magnetic Resonance Measurements in Patients with Left Ventricular Hypertrophy. *J Am Soc Echocardiogr* **28**, 1194-1203 (2015).

3. Wang, W.L., Zhou, Y.Q., Chai, N.N., Li, G.H., & Liu, D.W. Mediation and moderation analyses: exploring the complex pathways between hope and quality of life among patients with schizophrenia. *BMC Psychiatry* **20**, 22 (2020).

**Supplementary Tables**

**Supplementary Table S1. Baseline characteristics of the study participants according to sex.**

|  | Men  (N=2,218) | | Women  (N=1,962) | P-value |
| --- | --- | --- | --- | --- |
| Age, year | 71.0 (60.0-78.0) | | 76.0 (68.0-82.0) | <0.001 |
| BMI, kg/m² | 23.2 (20.9-25.6) | | 22.8 (20.2-25.7) | 0.005 |
| SBP, mmHg | 123 (109-142) | | 127 (110-145) | 0.003 |
| DBP, mmHg | 72.0 (62.0-83.0) | | 71.0 (62.0-82.0) | 0.819 |
| Heart rate, bpm | 85 (71-102) | | 86 (70-103) | 0.609 |
| NYHA class, n (%) |  | |  | 0.647 |
| Ⅰ/Ⅱ | 132 (8.7) | | 102 (7.6) |  |
| Ⅲ | 824 (54.8) | | 729 (54.4) |  |
| Ⅳ | 549 (36.5) | | 510 (38.0) |  |
| Past medical history, n (%) |  |  |  |  |
| Diabetes mellitus | 792 (35.7) | | 639 (32.6) | 0.036 |
| Hypertension | 1200 (54.1) | | 1195 (60.9) | <0.001 |
| IHD | 805 (36.3) | | 549 (28.0) | <0.001 |
| Atrial fibrillation | 631 (28.4) | | 599 (30.5) | 0.187 |
| Laboratory findings |  |  |  |  |
| TC, mg/dL | 146 (120-175) | | 154 (128-187) | <0.001 |
| Hemoglobin, g/L | 13.0 (11.0-14.4) | | 11.6 (10.2-13.0) | <0.001 |
| Sodium, mmol/L | 138 (134-140) | | 138 (134-140) | 0.546 |
| Potassium, mmol/L | 4.2 (3.8-4.6) | | 4.1 (3.7-4.6) | 0.003 |
| Troponin I, ng/mL | 0.1 (0.0-1.9) | | 0.1 (0.0-0.8) | <0.001 |
| AST, IU/L | 27.0 (19.0-43.0) | | 26.0 (19.0-40.0) | 0.006 |
| ALT, IU/L | 22.0 (13.0-38.0) | | 18.0 (11.0-30.0) | <0.001 |
| BUN, mg/dL | 22.0 (16.0-32.0) | | 20.0 (15.0-30.0) | <0.001 |
| Creatinine, mg/dL | 1.2 (0.9-1.7) | | 0.9 (0.7-1.4) | <0.001 |
| GFR, mL/min/1.73m^2^ | 62.2 (39.9-83.3) | | 59.6 (37.1-81.9) | 0.032 |
| HbA1c, %* | 6.2 (5.7-7.1) | | 6.1 (5.7-7.0) | 0.333 |
| Presentation glucose  level, mg/dL | 127 (103-172) | | 129 (104-172) | 0.645 |
| NT-proBNP, pg/mL | 4339 (1702-10416) | | 4970 (1825-12396) | 0.020 |
| Medication, n (%) |  |  |  |  |
| Beta blockers | 1349 (60.8) | | 1202 (61.3) | 0.992 |
| RAS blockers | 1543 (69.6) | | 1314 (67.0) | 0.028 |
| Spironolactone | 978 (44.1) | | 899 (45.8) | 0.394 |
| Diuretics | 1568 (70.7) | | 1475 (75.2) | 0.006 |
| Statin | 1211 (54.6) | | 1024 (52.2) | 0.034 |
| Echocardiographic parameters |  |  | |  |
| LVEDD, mm | 55.5 (50.0-62.0) | | 50.0 (44.8-55.6) | <0.001 |
| LVESD, mm | 44.0 (36.0-52.0) | | 36.0 (29.0-44.0) | <0.001 |
| LVEDV, mL | 130 (96-175) | | 88 (63-121) | <0.001 |
| LVESV, mL | 85 (52-126) | | 48 (28-82) | <0.001 |
| LV-EF, % | 34.2 (25.0-48.8) | | 44.7 (31.0-57.4) | <0.001 |
| HFpEF, n (%) | 514 (23.2) | | 783 (39.9) | <0.001 |
| LA diameter, mm | 45.0 (39.3-51.5) | | 43.3 (38.0-49.5) | <0.001 |
| LA volume, mL | 84 (62-115) | | 82 (59-113) | 0.064 |
| LAVI, mL/m^2^ | 48.9 (36.4-67.0) | | 54.8 (39.4-74.6) | <0.001 |
| E wave, m/s | 0.8 (0.6-1.1) | | 0.9 (0.6-1.1) | <0.001 |
| A wave, m/s | 0.7 (0.5-0.9) | | 0.8 (0.6-1.0) | <0.001 |
| Deceleration time, s | 158 (125-205) | | 169 (135-227) | <0.001 |
| E/e’ ratio | 15.7 (11.0-22.4) | | 17.3 (12.5-24.0) | <0.001 |
| Septum, mm | 10.2 (9.0-12.0) | | 10.0 (9.0-11.2) | <0.001 |
| Posterior wall, mm | 10.0 (9.0-11.5) | | 10.0 (9.0-11.0) | <0.001 |
| LVMI, g/m² | 133 (107-162) | | 123 (100-150) | <0.001 |
| RWT | 0.4 (0.3-0.4) | | 0.4 (0.3-0.5) | <0.001 |
| LVH, n (%) | 1308 (59.0) | | 1379 (70.3) | <0.001 |
| LVH type, n(%) |  | |  | <0.001 |
| Concentric LVH | 390 (29.8) | | 562 (40.8) |  |
| Eccentric LVH | 918 (70.2) | | 817 (59.2) |  |
| LV-GLS, % | 9.6 (6.5-13.3) | | 10.9 (7.7-14.9) | <0.001 |
| RV FAC, % | 37.0 (25.0-47.4) | | 39.2 (28.0-50.0) | <0.001 |

Values given as number (percentage), or median (interquartile range) unless otherwise indicated.

*HbA1c data was available in 42.3% patients.

ALT=alanine aminotransferase; AST=aspartate aminotransferase; BMI=body mass index; BUN=blood urea nitrogen; DBP=diastolic blood pressure; DM=diabetes mellitus; GFR=glomerular filtration rate; HbA1c=glycated hemoglobin; HFpEF, heart failure with preserved ejection fraction; IHD=ischemic heart disease; LA, left atrium; LAVI, LA volume index; LV, left ventricle; LVEDD, LV end-diastolic diameter; LVEDV, LV end-diastolic volume; LV-EF, LV ejection fraction; LVESD, LV end-systolic diameter; LVESV, LV end-systolic volume; LV-GLS, LV global longitudinal strain; LVH, LV hypertrophy; LVMI, LV mass index; NT-proBNP=N-terminal pro-brain natriuretic peptide; NYHA=New York Heart Association; RAS=renin-angiotensin system; RV-FAC, right ventricle fractional area change; RWT, relative wall thickness; SBP=systolic blood pressure; TC=total cholesterol.

**Supplementary Table S2. The statistical fit of structural equation models in the current study.**

|  | SEM in men | SEM in women | Standard criteria |
| --- | --- | --- | --- |
| CFI | 1.000 | 0.986 | >0.90 |
| RMSEA | 0.000 | 0.033 | <0.08 |
| SRMSR | 0.003 | 0.013 | <0.06 |

CFI, comparative fit index; RMSEA, root mean square error of approximation; SEM, structural equation modeling; SRMSR, standardized root mean square residual.

**Supplementary Table S3. Structural equation path models of direct and indirect effects of presentation glucose level/HbA1c for mortality.**

| Causal paths | | | | | | | Total  effects | | Direct  effects | Indirect  effects | | Standard Error | P-value |
| --- | --- | --- | --- | --- | --- | --- | --- | --- | --- | --- | --- | --- | --- |
| ***Presentation glucose level*** | | | | | | | |  | | |  | |  |
| Men | |  | | | |  | 0.095 | |  |  | | 0.021 | <0.001 |
| Glucose | → | |  | → | Mortality | |  | | 0.073 |  | | 0.021 | 0.001 |
| Glucose | → | | IHD | → | Mortality | |  | |  | 0.010 | | 0.003 | 0.001 |
| Glucose | → | | LV-GLS | → | Mortality | |  | |  | 0.012 | | 0.003 | <0.001 |
| Women | |  | | | |  | 0.106 | |  |  | | 0.022 | <0.001 |
| Glucose | → | |  | → | Mortality | |  | | 0.084 |  | | 0.023 | <0.001 |
| Glucose | → | | IHD | → | Mortality | |  | |  | 0.000 | | 0.004 | 0.970 |
| Glucose | → | | LV-GLS | → | Mortality | |  | |  | 0.022 | | 0.005 | <0.001 |
| ***HbA1c*** | |  | | | |  |  | |  |  | |  |  |
| Men | |  | | | |  | 0.025 | |  |  | | 0.032 | 0.429 |
| HbA1c | → | |  | → | Mortality | |  | | 0.000 |  | | 0.032 | 0.994 |
| HbA1c | → | | IHD | → | Mortality | |  | |  | 0.015 | | 0.006 | 0.007 |
| HbA1c | → | | LV-GLS | → | Mortality | |  | |  | 0.010 | | 0.005 | 0.026 |
| Women | | | | | | | 0.109 | |  |  | | 0.035 | 0.002 |
| HbA1c | → | |  | → | Mortality | |  | | 0.097 |  | | 0.035 | 0.005 |
| HbA1c | → | | IHD | → | Mortality | |  | |  | -0.002 | | 0.003 | 0.568 |
| HbA1c | → | | LV-GLS | → | Mortality | |  | |  | 0.014 | | 0.007 | 0.033 |

SEM models were constructed to identify the direct and indirect effects of presentation glucose level/HbA1c on the mortality. 5-year death data was used for mortality.

DM=diabetes mellitus; HbA1c=glycated hemoglobin; IHD=ischemic heart disease; LV-GLS=left ventricular global longitudinal strain; SEM=structural equation modeling.

**Supplementary Table S4. Structural equation path models of direct and indirect effects of DM for mortality, with an additional 3^rd^ indirect path of E/e’.**

| Causal paths | | | | | | | Total  effects | Direct  effects | Indirect  effects | Standard Error | P-value |
| --- | --- | --- | --- | --- | --- | --- | --- | --- | --- | --- | --- |
| Men | |  | | | |  | 0.065 |  |  | 0.024 | 0.007 |
| DM | → | |  | → | Mortality | |  | 0.042 |  | 0.024 | 0.087 |
| DM | → | | IHD | → | Mortality | |  |  | 0.013 | 0.005 | 0.011 |
| DM | → | | LV-GLS | → | Mortality | |  |  | 0.006 | 0.003 | 0.081 |
| DM | → | | E/e’ | → | Mortality | |  |  | 0.005 | 0.003 | 0.053 |
| Women | |  | | | |  | 0.088 |  |  | 0.025 | <0.001 |
| DM | → | |  | → | Mortality | |  | 0.067 |  | 0.026 | 0.009 |
| DM | → | | IHD | → | Mortality | |  |  | -0.001 | 0.005 | 0.806 |
| DM | → | | LV-GLS | → | Mortality | |  |  | 0.012 | 0.004 | 0.004 |
| DM | → | | E/e’ | → | Mortality | |  |  | 0.011 | 0.004 | 0.003 |

SEM models were constructed to identify the direct and indirect effects of DM on the mortality. 5-year death data was used for mortality.

DM=diabetes mellitus; HbA1c=glycated hemoglobin; IHD=ischemic heart disease; LV-GLS=left ventricular global longitudinal strain; SEM=structural equation modeling.

**Supplementary Figures Legends**

**Supplementary Figure S1. Kaplan-Meier curves for 5-year all-cause mortality according to HbA1c levels in men and women.**

Kaplan-Meier survival curves according to HbA1c levels (HbA1c <5.7%; 5.7 ≤ HbA1c <7.0%; and HbA1c ≥7.0%) in men and women.

DM=diabetes mellitus; HbA1c=glycated hemoglobin.

**Supplementary Figure S2. Sex-specific association of presentation glucose level with E/e’ ratio.**

RCS curves were plotted between presentation glucose level and E/e’ ratio. Each dot indicates an individual patient’s data.

RCS=restricted cubic spline

**Supplementary Figure S3. Path diagrams of relationship between presentation glucose level/HbA1c and mortality according to sex.**

Diagrams of the structural equation model using presentation glucose level (A) and HbA1c (B) for independent variables in men and women. Standardized path coefficients are shown indicated on each path as effect estimates. Solid lines denote significant paths, and dashed lines denote non-significant paths. The 5-year mortality data were used in this model.

HbA1c=glycated hemoglobin; IHD=ischemic heart disease; LV-GLS=left ventricular global longitudinal strain

*P-value <0.05, **P-value <0.001

**Supplementary Figure S4. Path diagrams of relationship between DM and mortality according to sex, with an additional 3^rd^ indirect path of E/e’.**

Diagrams of the structural equation model using IHD, LV-GLS, and E/e’ ratio as mediators linking DM and mortality in men and women. Standardized path coefficients are shown indicated on each path as effect estimates. Solid lines denote significant paths, and dashed lines denote non-significant paths. The 5-year mortality data were used in this model.

DM=diabetes mellitus; HbA1c=glycated hemoglobin; IHD=ischemic heart disease; LV-GLS=left ventricular global longitudinal strain

*P-value <0.05, **P-value <0.001

**Supplementary Figure S5. Correlation between presentation glucose level and HbA1c level.**

Scatter plots of presentation glucose level and HbA1c value in men and women. Each dot indicates an individual patient’s data. Linear regression line (solid line) and 95% confidence interval (shaded area) is depicted. Presentation glucose level had a moderate correlation with HbA1c value in both sexes. (Pearson's correlation coefficient (r)=0.55, p<0.001 for men; r=0.45, p<0.001 for women)

HbA1c=glycated hemoglobin

**Supplementary Figures**

**Supplementary Figure S1**

**
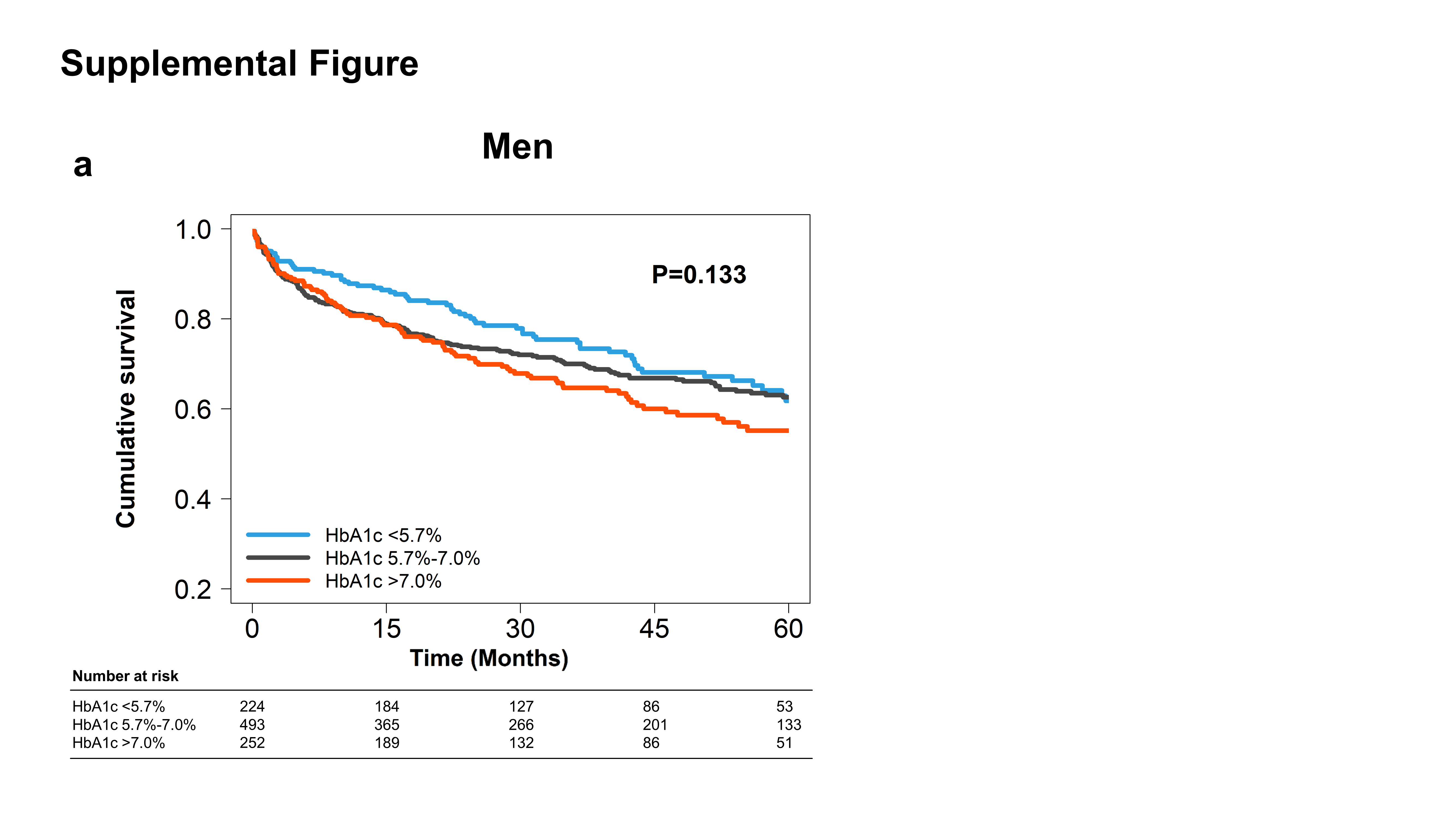
**


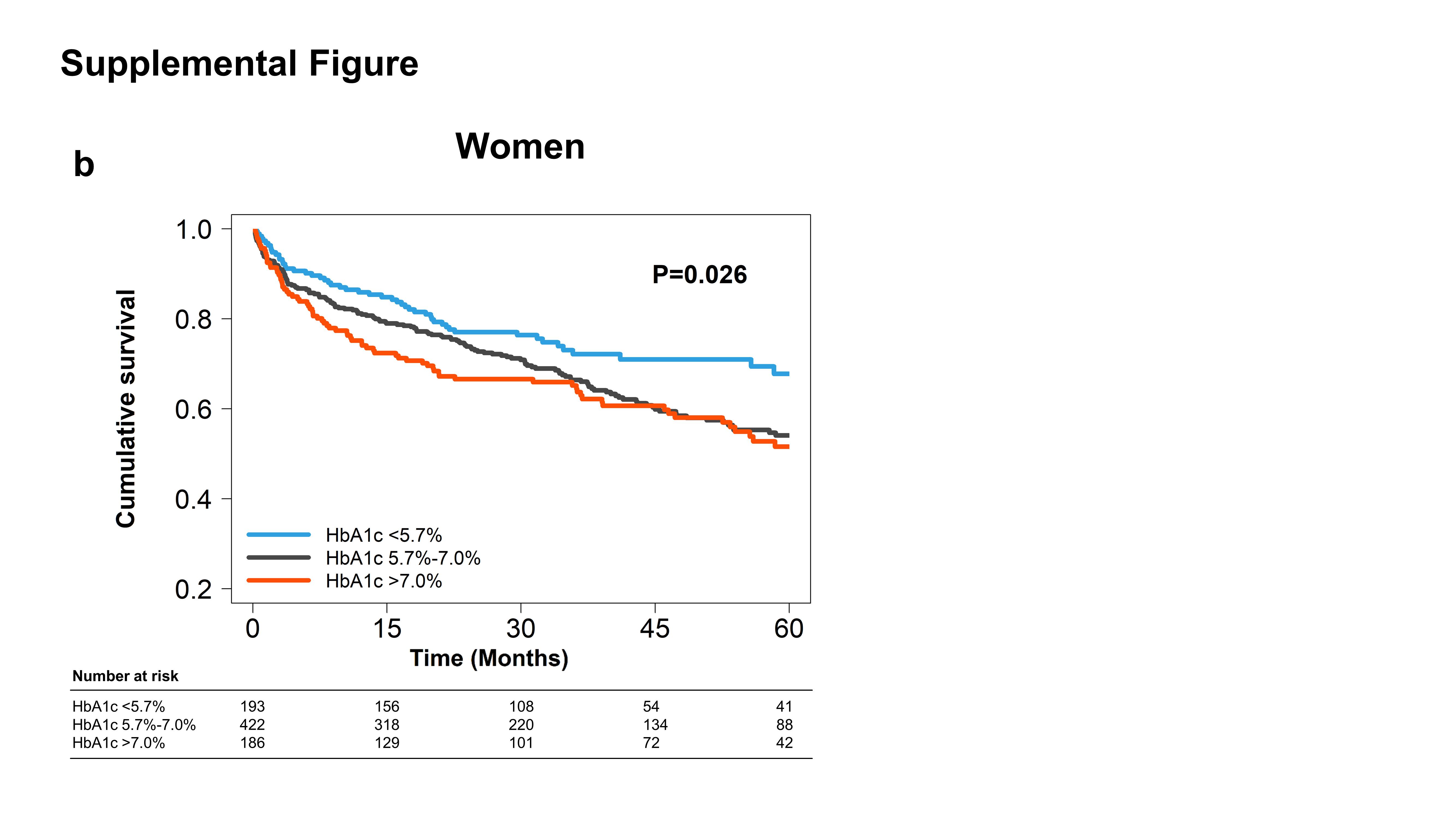


**Supplementary Figure S2**


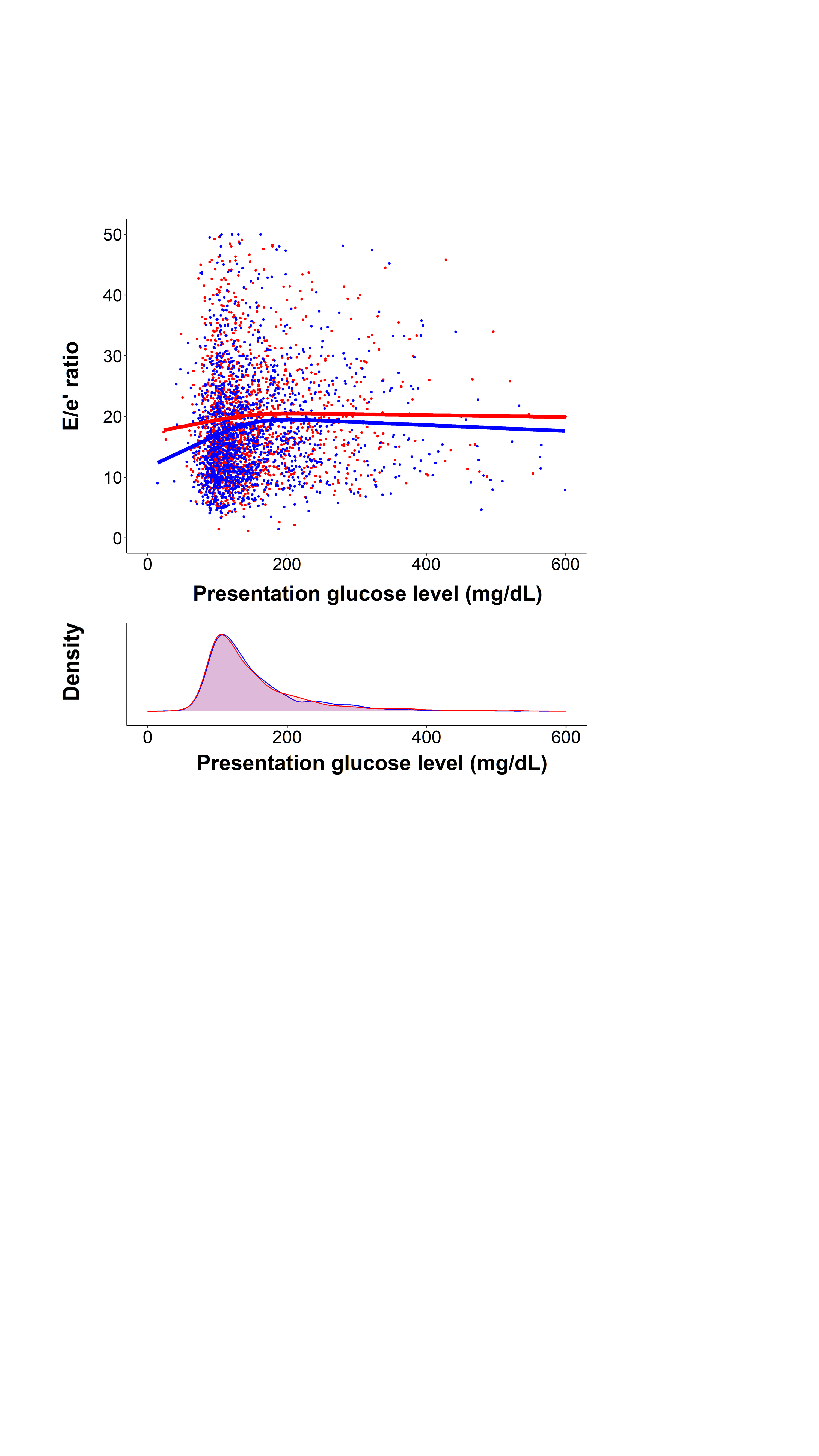


**Supplementary Figure S3**


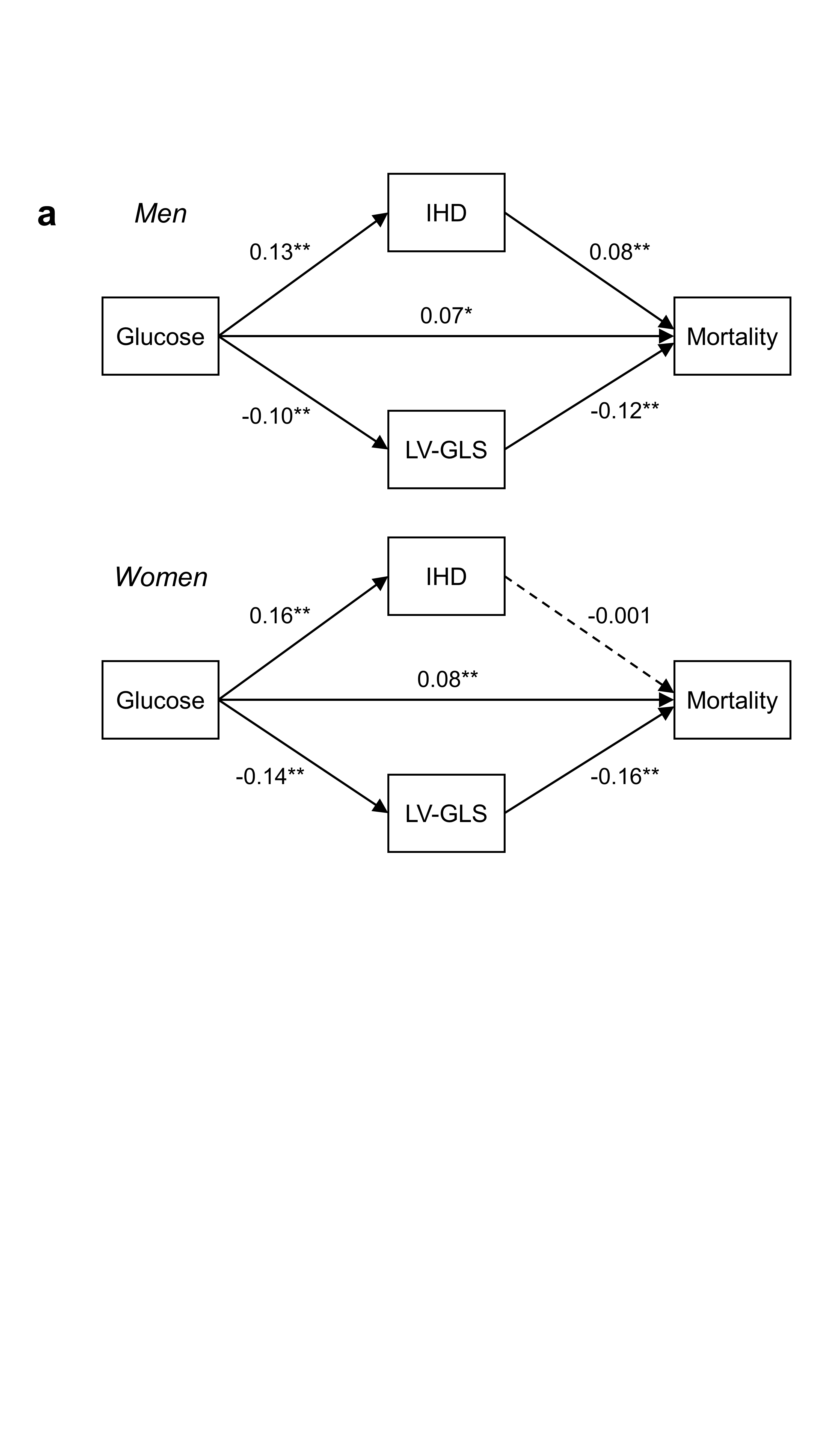

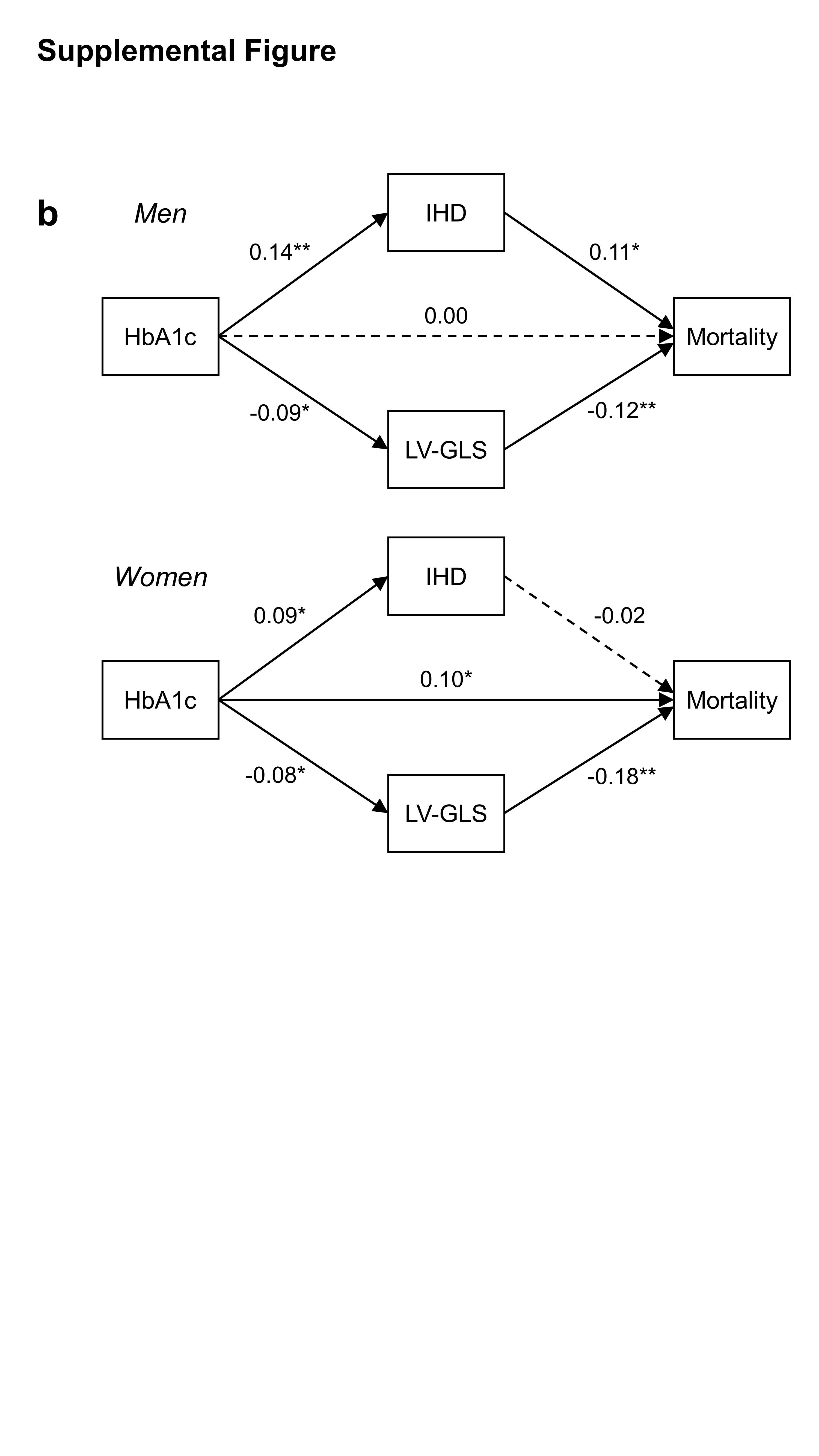


**Supplementary Figure S4**


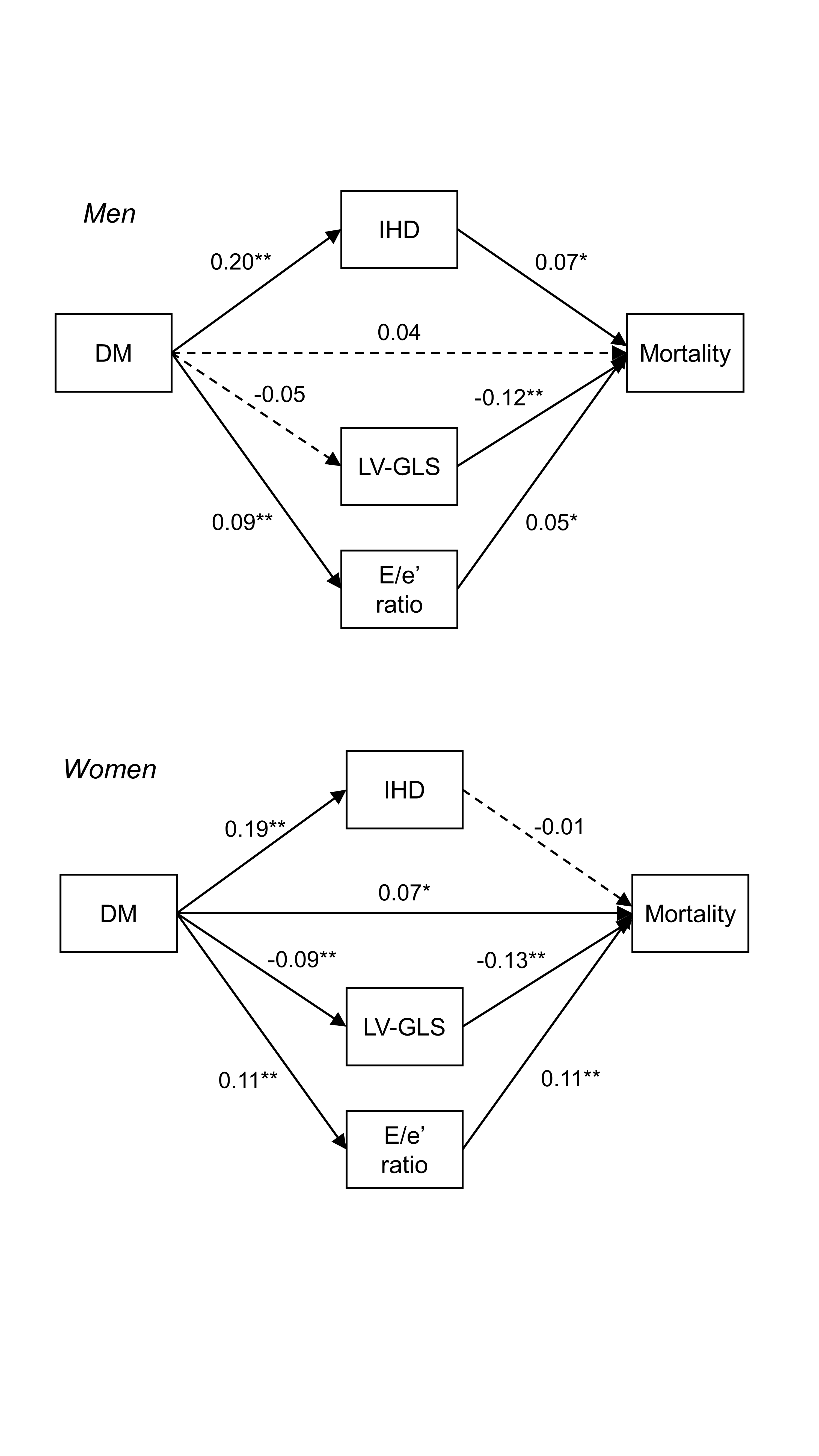


**Supplementary Figure S5**

**
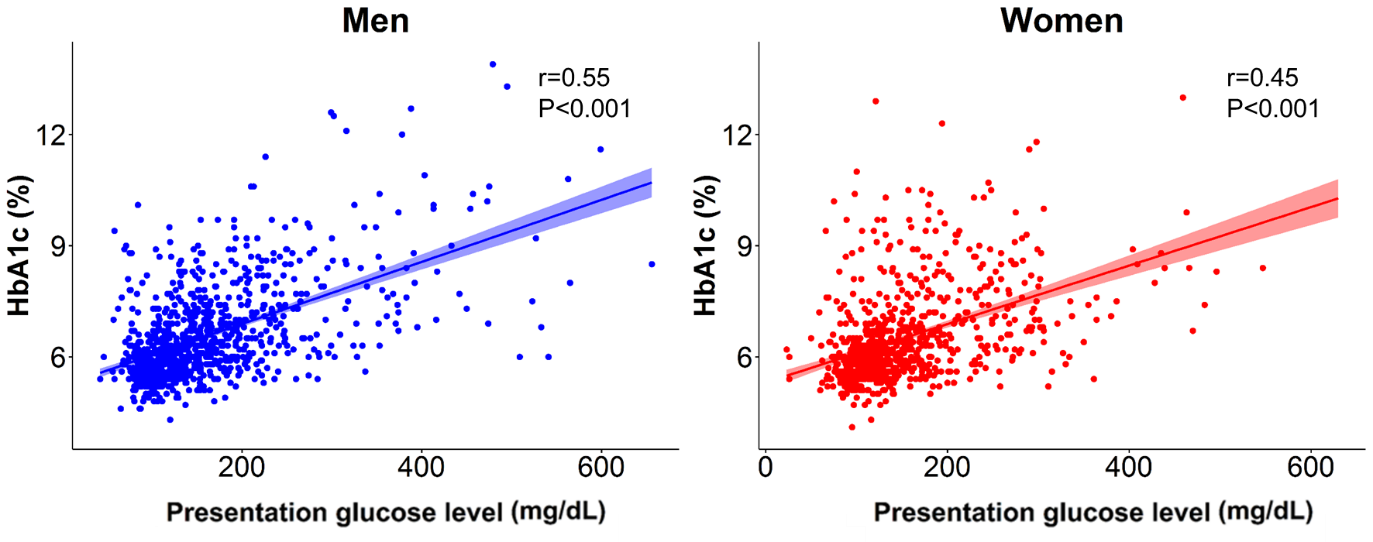
**
